# Readmission to the ICU: is it a big concern? An analysis

**DOI:** 10.1186/cc14643

**Published:** 2015-03-16

**Authors:** A Ahmed, A Datta, A Kar

**Affiliations:** 1 Medica Superspecialty Hospital, Kolkata, India

## Introduction

Readmission to the ICU is an important quality indicator of ICU care. We conducted a prospective study in a level 3 care ICU in Kolkata of a tertiary care hospital to analyze whether there are overall outcome differences when comparing the readmission group with the entire group.

## Methods

Our prospective study included 2,140 patients admitted to a level 3 care ICU over a period of 1 year. The number of readmissions (*n = *85) during the same period was also documented. Readmission was defined as all patients who were transferred back to the ICU prior to hospital death/discharge during the above period. ICU outcome was calculated using the predictive APACHE IV model. Payment methods were documented as either self-paying or corporate/insurance paying. A comparison analysis between the entire group with the readmission group was done using unpaired Student *t *test and *P *< 0.05 was considered statistically significant.

## Results

In the entire group (*n = *2,140), mean APACHE IV was 50.34 ± 31.54 SD (median 42), PMR 15.49, observed deaths 327, ALOS 4.05 days ± 4.55 SD (median 3), SMR 0.99 (CI = 0.88 to 1.1), mean age 60.55 years ± 15.68 SD (median 63), 490 ventilations, 72.71% of patients were self-paying while 27.29% of patients were corporate/insurance paying. In the readmission group (*n = *85), mean APACHE IV was 77.16 ± 33.72 SD (median 73), PMR 38.89, observed deaths 42, ALOS 5.23 days ± 4.18 SD (median 4), SMR 1.27 (CI = 0.95 to 1.67), mean age 64.79 years ± 14.40 SD (median 67), 43 ventilations, 75.3% of patients were self-paying while 24.7% of patients were corporate/insurance paying. During comparison between the two groups there were statistically significant differences, with the readmission group having significantly higher APACHE IV (*P *< 0.0001), PMR (*P *< 0.0001), ALOS (*P *= 0.002), age (*P *= 0.005), and SMR (1.27 vs. 0.99) compared with the entire group. Percentage of patients requiring ventilation (50.59% vs. 22.90%) and mortality rate (49.11% vs. 15.28%) were also significantly higher in the readmission group. Readmission was significantly higher in the self-paying group. Root-cause analysis showed most readmissions were due to deteriorating conditions (desaturation, hypotension, sepsis, arrhythmias); however, it was also associated with cases where transfer policy from the ICU was not followed by stakeholders and financial issues were a cause of early transfer.

## Conclusion

Readmission to the ICU was associated with worse outcome in our study group. Lack of adherence to transfer policy by concerned stakeholders was a concern as well as increasing cost of healthcare.

